# Empowering employees with chronic diseases; development of an intervention aimed at job retention and design of a randomised controlled trial

**DOI:** 10.1186/1472-6963-8-224

**Published:** 2008-11-04

**Authors:** Inge Varekamp, Gabe de Vries, Annelies Heutink, Frank JH van Dijk

**Affiliations:** 1 Coronel Institute of Occupational Health, Academic Medical Center, University of Amsterdam, The Netherlands; 2 Department of Psychiatry, Programme for Mood Disorders, Academic Medical Center, University of Amsterdam, Amsterdam, The Netherlands; 3 Arbo Unie Occupational Health Service, Amsterdam, The Netherlands

## Abstract

**Background:**

Persons with a chronic disease are less often employed than healthy persons. If employed, many of them experience problems at work. Therefore, we developed a training programme aimed at job retention. The objective of this paper is to describe this intervention and to present the design of a study to evaluate its effectiveness.

**Development and description of intervention:**

A systematic review, a needs assessment and discussions with Dutch experts led to a pilot group training, tested in a pilot study. The evaluation resulted in the development of a seven-session group training combined with three individual counselling sessions. The training is based on an empowerment perspective that aims to help individuals enhance knowledge, skills and self-awareness. These advances are deemed necessary for problem solving in three stages: exploration and clarification of work related problems, communication at the workplace, and development and implementation of solutions. Seven themes are discussed and practised in the group sessions: 1) Consequences of a chronic disease in the workplace, 2) Insight into feelings and thoughts about having a chronic disease, 3) Communication in daily work situations, 4) Facilities for disabled employees and work disability legislation, 5) How to stand up for oneself, 6) A plan to solve problems, 7) Follow-up.

**Methods:**

Participants are recruited via occupational health services, patient organisations, employers, and a yearly national conference on chronic diseases. They are eligible when they have a chronic physical medical condition, have a paid job, and experience problems at work. Workers on long-term, 100% sick leave that is expected to continue during the training are excluded. After filling in the baseline questionnaire, the participants are randomised to either the control or the intervention group. The control group will receive no care or care as usual. Post-test mail questionnaires will be sent after 4, 8, 12 and 24 months. Primary outcome measures are job retention, self efficacy, fatigue and work pleasure. Secondary outcome measures are work-related problems, sick leave, quality of life, acquired work accommodations, burnout, and several quality of work measures. A process evaluation will be conducted and satisfaction with the training, its components and the training methods will be assessed.

**Discussion:**

Many employees with a chronic condition experience problems in performing tasks and in managing social relations at work. We developed an innovative intervention that addresses practical as well as psychosocial problems. The results of the study will be relevant for employees, employers, occupational health professionals and human resource professionals (HRM).

**Trial registration:**

ISRCTN77240155

## Background

Persons with longstanding health problems or handicaps have paid jobs less often than healthy persons. The employment rate in several countries in Europe is approximately one third lower for these individuals [1-3]. These figures differ substantially for various chronic diseases. The majority of rheumatoid arthritis patients in the USA and the Netherlands are employed (59% and 56%), although the prevalence of premature work cessation rises steadily with disease duration [4,5]. For inflammatory bowel disease the figures are roughly the same: about 60% [6], or even more [7,8] are employed. For the USA the figures are somewhat higher, for Europe somewhat lower. In addition, patients with chronic obstructive pulmonary disease (COPD) perform rather well: for Dutch patients between the ages of 45–60, 52% are employed [9]. More dramatic are the figures for dialysis patients or people with Parkinson's disease, where less than one third of the patients of working age report being employed [10-12]. For multiple sclerosis patients, comparable figures are available: only 20 – 40% are employed [13].

If employed, many persons with chronic diseases experience problems at work. Lerner et al. [14] studied a large sample in the USA and concluded that, depending on the chronic disease, between 22% and 49% of the employees experienced difficulties in meeting physical work demands, and that between 27% and 58% had difficulty meeting psychosocial work requirements. Compared to healthy workers, chronically ill workers have higher scores on scales measuring fatigue and emotional exhaustion, which are correlated with perceived work stress [15,16]. Research focussing on the patients' perspectives provides insight into possible sources of stress and fatigue, and offers suggestions for remedies. Patients with diabetes, rheumatoid arthritis or hearing loss stated that important factors that helped them to continue working were the ability to cope with the illness, support from management and colleagues, and adequate work conditions [17]. A focus group study among employees with inflammatory arthritis reveals that they faced difficulties managing interpersonal and emotional difficulties at work, in addition to managing fatigue and other symptoms, and that they had trouble managing working conditions [18]. Asked what they expected in the way of work-related support, employees with multiple sclerosis mentioned support with managing work performance and support with managing social and personal expectations [19]. These findings suggest that vocational rehabilitation efforts should pay attention to psychosocial as well as practical bottlenecks at the workplace.

For the past several decades, social policy in many countries has been focussed on helping individuals with a chronic disease or handicap enter or re-enter the labour market, whereas less attention is paid to efforts aimed at helping employees to stay at work. Finding a new job is more difficult than trying to keep one, as one has the extra task of convincing a new employer of one's capabilities. This might be a reason to focus attention on structural vocational rehabilitation efforts aimed at job retention.

A systematic review shows that there is some evidence for the effectiveness of interventions of this kind. However, the number and methodological quality of the studies is not sufficient to tell which one will be most successful [20]. Based on this review and discussions with experts, we developed training for employees with chronic diseases that supports them in solving practical and psychosocial problems. The aim is to prevent the unnecessary loss of their job.

The objective of this article is twofold. First, the development, set-up and contents of the intervention will be described. Second, we will specify the design of the study to evaluate its effectiveness.

## Development and description of intervention

### Target group and purpose

This intervention is meant for employees with a chronic physical (i.e. not a predominant psychiatric) disease, who experience work-related problems and fear job loss or loss of work pleasure. We decided to include a wide variety of chronic diseases, such as musculoskeletal diseases like arthrosis and rheumatoid arthritis, neurological diseases like multiple sclerosis and Parkinson's disease. We included endocrinological diseases like diabetes, heart failure, pulmonary conditions, inflammatory bowel disease, chronic fatigue syndrome, and visual impairment, as well as any other chronic disease or handicap that results primarily in physical limitations. Work-related problems are broadly defined – they may be practical, social, mental or a combination of the three.

The aim of the intervention is twofold: job retention as well as maintenance or increase of work pleasure.

### Program development

We started to carry out a systematic review of vocational rehabilitation interventions aimed at job retention for employees with chronic diseases [20]. Effectiveness studies, though often of low methodological quality, gave evidence of positive effects. This inspired us to develop an intervention of the same kind. Four patient organisations were contacted to ask whether they thought that there was a need for this kind of intervention. Three employees with chronic diseases who had experienced serious work-related problems were interviewed by telephone in order to assess their needs. A first draft of a program was developed, based on international examples. In addition, elements of the program were derived from two current Dutch vocational rehabilitation programs aimed at job retention for employees on long-lasting sick leave. One is tailored to workers with burnout [21], the other to workers with severe depression [22,23]. The pilot version of the training was tested in a group of eight employees. On the basis of the trainers' experiences, the researchers' observations, a pre- and post test evaluation and an interview of the participants by telephone, the pilot version was adapted. In the process of adaptation, decisions were reached about the optimum length of the training period. Elements of the pilot training were prioritised, which resulted in the elimination of several elements.

The most important post-pilot changes included a new final meeting, two months after the sixth meeting. More time was reserved for role-playing, and two individual consultations were added to the first intake consultation. A 'Quality of work' model, used to clarify work-related problems and based on the ICF disability model [24], was not helpful in clarifying work related problems, because many problems experienced at work originated in 'the environment', a concept that is present but not elaborated well in the ICF. Therefore, this model was substituted for a new version that emphasises the positive or negative influence of work tasks, social relationships at the workplace and working conditions on wellbeing at work.

After the decision-making process on the outlines was finished, the essential elements, procedures and objectives of each component of the group sessions, as well as of the individual counselling sessions, were discussed and described in detail in the trainers' manual. Together with the trainers' manual, a textbook for the participants was written. This textbook gives an overview of the content of every group session, homework to be completed for the next session and an appendix that offers theoretical background and exercises. Experts from two patients' associations commented on the training and the textbook.

### Rationale of the training

The training is based on a number of notions:

#### Empowerment

Participants are invited to participate in a program 'to provide knowledge, skills and a heightened self-awareness regarding values and needs, so that patients can define and achieve their own goals', corresponding to the definition of empowerment by Feste and Anderson [25]. Such a program requires an active attitude, in which participants define what is problematic at work and subsequently try to get a hold on their situation. Counselling can be a component of such an empowerment program.

#### The importance of personal and environmental factors

Work-related problems and work disability can be understood as the result of the specific combination of disease, person and workplace. A serious medical condition can be decisive; causing so many problems that continuing work is impossible. On the other hand, whether an employee with a chronic disease becomes work disabled often depends on factors other than the severity of his disease or bodily impairments. The actual disability may depend on personal and environmental factors that can hinder or promote work capacity and functioning. This point of departure corresponds well with the WHO's International Classification of Functioning, Disability and Health [24,26]. However, the ICF-model is not elaborate enough to serve as a model to clarify work-related problems. These must be understood in a broader context in which work tasks, social relationships at the workplace, working conditions and terms of employment are understood as significant for well-being at work.

#### Communication is important and can be difficult

Working together and discussing tasks and responsibilities requires communication skills. However, having a chronic disease may hamper communication and have a negative impact on social relationships with supervisors and colleagues. Employees need to explain to the supervisor or colleagues what their disease implies and to elucidate its consequences for work performance. At the same time feelings of sadness, shame or anger about their disease may prevent speaking out [27]. Not speaking out or non-assertive behaviour is an impediment to the solution of work-related problems

#### Perceived self-efficacy is a prerequisite to resolving work-related problems

According to social learning theory, active coping behaviour aimed at solving problems will improve when perceived self-efficacy increases [28,29]. Expectations of personal efficacy will be enhanced by performance accomplishments, vicarious experience and verbal persuasion.

The above-mentioned principles resulted in the development of a stepwise intervention for employees with a chronic disease: a) exploring and clarifying work-related problems, b) communication at work, and c) thinking out and realising solutions. It is organised mainly as a group intervention, since group meetings are a suitable method for enhancing perceived self-efficacy.

### Set-up of the training

The training is a group training consisting of seven three-hour sessions every two weeks. The last session takes place two month after the sixth session. The group comprises eight participants and one trainer. The trainer is experienced in working with groups, has psycho-therapeutic knowledge of the principles of rational emotive therapy as well as knowledge of occupational psychology and a basic understanding of chronic diseases and their consequences.

Participants are requested to read material from the textbook before each session, and to do homework that is discussed at the start of the following session. The exchange of experiences forms an important part of the training. Guest speakers are invited at three sessions. An actor is invited twice to assist with role-playing. An occupational physician and an employment expert are invited to discuss matters concerning work accommodations, sickness absence, disability pensions and other practical topics. In conjunction with the group sessions three individual consultations are offered: one at the beginning, one halfway through the training, and one after the sixth session. These consultations offer the trainer the possibility of giving feedback, and participants the possibility of discussing anything they want in private, or to pursue questions in greater depth.

### Contents

Every session focuses on one theme, which will be discussed briefly.

#### 1. What bothers you; consequences of a chronic disease in the workplace

The participants get to know each other well in this session; group dynamics and the feeling that one can exchange experiences and practice exercises safely are essential for the success of the training. Attention is paid to possible consequences of chronic diseases in terms of difficulties in performing tasks, in carrying on, and in the risk of sickness absence or work disability.

**Figure 1:**
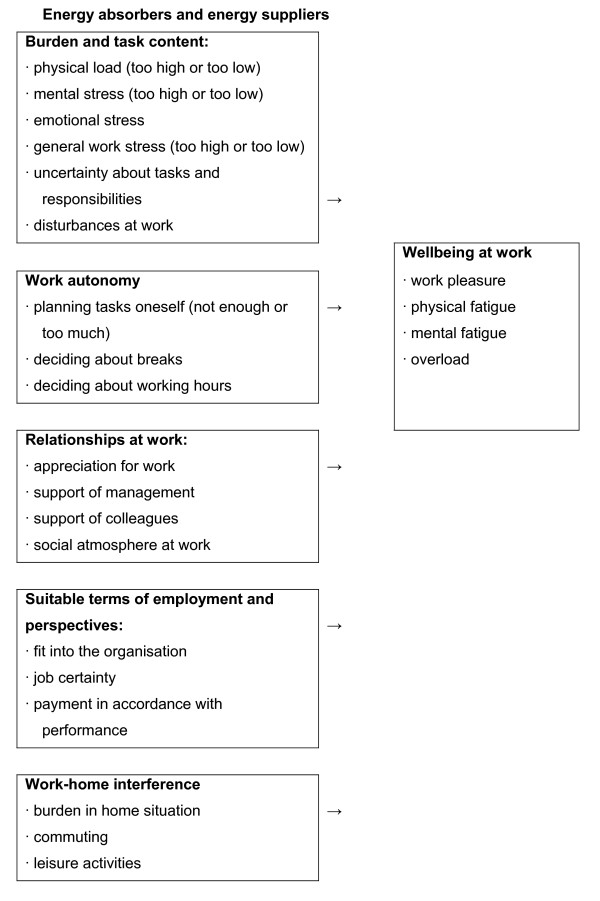
Model 'Quality of work'.

The 'Quality of work' model is used to explore work-related problems (figure 1). This model contains groups of factors that are known for their influence on quality of work. It is based on theoretical ideas about work demands and work capacity [30], research on employees with chronic diseases, and recent views developed in occupational psychology on work factors that yield or absorb energy [31]. It is explained that, for some factors, it holds that not only 'too high' or 'too much', but also 'too low' may be problematic. For instance a high mental burden can be as problematic as monotonous work without any mental challenge.

Two participants are asked to explore the negative and positive factors of their work in the group. They do so with the help of a large laminated poster of the 'Quality of work' model in which plus signs or minus signs show aspects of their work that they experience as positive or negative. The others are asked to fill in the model for the next sessions. The input of all participants will be discussed extensively in the group at least at one session.

#### 2. Insight into yourself: feelings and thoughts about having a chronic disease

Persons with a chronic disease experience that talking about one's disease or consulting about work accomodations with a supervisor require good communication skills. However, negative thoughts or feelings about the disease can be an obstacle. Feelings of sadness or shame and thoughts of worthlessness can lead to non-assertive behaviour. Feelings of anger may induce aggressive verbal behaviour. The purpose of this meeting is to explore feelings and thoughts. The intention is not to replace them, but to understand how these feelings and thoughts might affect coping behaviour and might lead to ineffective communication. Homework for this session is to formulate predominant thoughts around work and illness. A second task is to request a consultation with the supervisor, to discuss how he or she appreciates the job performance. This is regarded as a preparatory consultation; a following consultation will be about concrete problems and solutions.

#### 3. Communication: practicing in daily work situations

Employees with a chronic disease do not always stand up for themselves. The actor in this session shows what the difference is between non-assertive, assertive and aggresive verbal behaviour. This is followed by a role playing exercise; the participants explain their chronic disease to 'a new colleague', and talk about what consequences it has for daily functioning at the workplace, why this colleague should know about it, and how they would like the colleague to deal with it. The other participants give feedback.

#### 4. Practical matters; the occupational physician, the employment expert, legislation and facilities for disabled employees

The textbook gives an overview of the occupational physicians' function, as well as legislation concerning sickness absence and work disability. Furthermore, work accommodations and other facilities for disabled employees or their employers are listed. By way of homework every participant formulates one question for the occupational physicain and one question for the employment expert on matters that are relevant to themselves. The guest speakers have received these questions beforehand and discuss them in the group. Homework for the following session is to consider which work accommodations might be appropriate, and to initiate a second consultation with the supervisor about work-related problems and solutions. If appropriate, a consultation with the occupational physician of the company is recommended.

#### 5. Communication and standing up for oneself: continuation

Examples and theorising about short-term and long-term functions of different manifestations of verbal behaviour are given to deepen understanding of assertive, non-assertive, and aggressive behaviour. Subsequently, the participants practice with the actor situations they find difficult at work, for instance negotiations with their supervisor or conversations in which they deal with their colleagues' lack of understanding.

#### 6. A plan to solve problems

The homework for this session is to develop a plan to tackle one or more of the resulting work-related problems. This plan is developed along SMART-lines: Specific, Measurable, Acceptable, Realistic, and Time specific. The plans are discussed in small groups and adapted if necessary.

#### 7. Follow-up: what works and what not?

The last session is meant as a follow-up meeting. Experiences with the implementation of the plan are discussed. By way of conclusion the participants write a letter to themselves, in which they describe how far they have gotten and what they want to have achieved in a half year's time. This letter is meant to keep them active and will be sent a half year later.

## Methods/Design

### Study design, research question, and follow-up

The study is designed as a randomised controlled trial. Eight training groups, with 64 participants in total, will be compared to about 64 persons in the control group. The follow-up is two years, with one baseline questionnaire and four follow-up questionnaires at 4, 8, 12 and 24 months.

The research question is twofold: a) Which work-related problems do employees with a chronic disease experience at the workplace, b) Does participation in the training increase self efficacy, establish work accommodations, decrease fatigue, enhance work pleasure, improve quality of work, and contribute to job retention?

Persons in the control group receive care as usual. However, the usual care for this group of patients for work-related issues varies from nothing at all to counselling or support by occupational health professionals or medical professionals from outpatient clinics.

The Medical Ethics Committee of Academic Medical Center in Amsterdam informally approved of the study idea, but deemed ethical review unnecessary because they perceived no question of 'medical' research.

### Inclusion criteria

Participants are eligible for the study when they have a chronic physical disease, have a paid job, experience problems at work, fear losing their job or job satisfaction, and are willing to undertake actions to solve problems. Workers with predominant psychiatric conditions are excluded; people with a chronic physical disease in combination with depressive feelings are not excluded. Workers on long-term 100% sick leave that is expected to continue during the training are excluded.

### Recruitment of participants

Participants are recruited via outpatient clinics, occupational health services, patient organisations, employers, and a yearly national conference on chronic diseases. Presentations are given at outpatient clinics and occupational health care services; specialised nurses, medical specialists and occupational physicians are asked to draw attention to the project by offering potential participants a leaflet. The leaflet is also available digitally. Patients' organizations are asked to publish calls for participation in their magazines, electronic newsletters and websites. A mailing is sent to a large number of employers, who publish calls for participation in house organs or approach potential participants directly. Presentations are given for meetings of patient organizations. Potential participants or medical professionals have the possibility to ask for information by mail or telephone.

The training is offered for free eight times in the course of one and a half years.

### Organisation of enrolment

Candidates apply by telephone. They can not be presented by others, (e.g. medical professionals). A first check at the moment of registration is on the objective inclusion criteria: chronic physical disease, paid job, and no long-term full-time sick leave. Candidates receive a written confirmation of their registration, explaining the procedures. Candidates receive the baseline questionnaire and the informed consent form three weeks before the randomisation. After a first and a second reminder, all participants who have returned the questionnaire are randomised.

### Randomisation

Since not all questionnaires will be returned, the ideal group size is 18. If four or more persons have the same disease, randomisation is stratified on this disease, in order to prevent a coincidentally large group within the training group that shares the same disease. Randomisation is performed by the researcher in the company of another person, and with help of a computer program generating random numbers. Since ethical considerations preclude individual consultation before randomisation, persons randomised in the training group receive the invitation for a first individual consultation afterwards. If the trainer or the participant decide that the program does not meet the participants' expectations, a new randomisation procedure starts with the remaining persons in the control group.

### Outcome measures

Primary outcome measures are job retention, self efficacy, fatigue and work pleasure.

Not having a paid job, or having more than six months full-time sick leave in combination with the expectation that return to work is impossible or improbable is considered as job loss.

Self-efficacy is measured by a situation-specific instrument, measuring self-efficacy in solving work and disease related problems. It is developed according to the principles formulated by Bandura [32]. The fourteen items are measured on bipolar five-point Likert scales.

Fatigue is measured with the Checklist Individual Strength (CIS), a well-validated questionnaire for the working population [33]. It has four subscales: fatigue severity (8 items), concentration (5 items), motivation (4 items) and physical activity level (3 items).

Work pleasure is measured with a subscale of the Dutch questionnaire on Perception and Judgement of Work [34].

Secondary outcome measures are work-related problems, sick leave, quality of life, acquired work accommodations, burnout, and three quality of work measures: social relationships with colleagues and supervisor, and worries about work. These are subscales of the Dutch questionnaire on Perception and Judgement of Work [34].

Work-related problems are measured with eight items: having problems with specific work tasks, finishing work, arranging the workplace, commuting, communicating with colleagues, communicating with supervisors, accepting the disease, and balancing work and life at home. The three answer categories are counted as 0 (no), 1 (yes, slightly) or 2 (yes, severely) and are added up to an index measure.

Sick leave is measured as the number of days on sick leave during the last four months.

Quality of life is measured with the validated SF12 [35].

Work accommodations are measured with the Work Accommodations List [9].

Burnout is measured by the Utrecht Burnout Scale (UBOS) [36].

### Sample size and power

The sample size is based on detecting a difference in fatigue, measured with the fatigue severity subscale of the Checklist Individual Strength (CIS). Power calculations have been made with an alpha of 0.05 and a power of 80%. Studies examining the effect of interventions on fatigue of persons with a chronic disease are rare. Stulemeijer et al (2005) studied the effect of cognitive behaviour therapy for adolescents with chronic fatigue syndrome in a randomized controlled trial. In the treatment group fatigue severity decreased from 52.5 to 30.2 (sd = 16.8), compared to a decrease from 51.6 to 44.0 for the control group [37]. Based on these figures, we need 25 persons in the intervention group and the control group each.

Our intervention resembles Stulemeijer's intervention in its aim to decrease fatigue. However, whereas Stulemeijer's intervention focused on the disease and its symptoms, our empowerment training for employees focuses on work related problems and resulting work stress and fatigue. The chronic disease itself will remain and might even progress, which means that fatigue levels comparable with those of the healthy workforce are not to be expected at follow-up. This is the reason why we have chosen a larger sample, of 64 persons in the intervention group and the control group each.

### Statistical analysis

Statistical analyses will be performed according to the intention-to-treat principle. Job retention will be analysed using survival analysis. The other variables will be analysed with repeated measurement analysis and mixed linear models.

### Process evaluation

The process of the intervention will be evaluated in three ways. First, we will describe the recruitment of participants and evaluate if we reached the target group and if our recruitment methods worked or failed. The researcher keeps a recruitment diary for this purpose. Second, the group sessions and the individual sessions will be evaluated by the trainers. They fill in a process evaluation form and note the attendance of the participants, whether each subject for that session has been discussed, whether the participants experience emotional or cognitive difficulties with the subject, whether they feel involved, and whether the goal of the specific subject is reached. Third, the participants are asked their opinion in the post-test questionnaires. They are asked to evaluate the whole training, the various themes and procedures, and the textbook. They are also asked to evaluate whether skills they wanted to improve have actually improved, whether they have passed successfully through the three stages: clarification, communication and problem solution, and whether they have attained the goal they had in mind beforehand.

## Discussion

Vocational rehabilitation interventions for persons with chronic diseases generally focus on entering or re-entering the labour market. Structured vocational rehabilitation interventions aimed at job retention are rare, notwithstanding demands for evidence-based vocational rehabilitation programmes aimed at preventing work disability for this group of employees [38]. Just a few of this kind of interventions could be traced in a systematic review [20]. A reason for this lack of initiatives or lack of documentation and evaluation might be that the societal consequences of work related problems are not felt clearly as long as people are still struggling to retain their jobs. However, when serious problems in work functioning finally result in long-term sickness absence, complete work disability or loss of a paid job, it is difficult to return to work.

The intervention we developed originates from an empowerment perspective and aims to help employees restore the balance of work capacity and work demands. We used a stepwise approach, starting with exploring practical, psychological or social problems, followed by communicating with the supervisor or others at work, and finally developing and implementing solutions.

Most studies on interventions aimed at job retention claim effectiveness. However, these claims are seldom underpinned with a study design offering strong evidence. Studies seldom use pretesting, a control group, a sufficient number of participants or a long-term follow-up. Our study design involves a control group and outcome assessments at five points over two years. We also try to include 128 participants randomised over two conditions.

An inevitable drawback is that participants are not blinded. The research project may trigger the awareness of the problems of participants, which can result in more than usual active coping behaviour in members of the control group.

The results of this study will generate knowledge about the nature of work-related problems and will possibly contribute to better vocational rehabilitation services for employees with chronic diseases. It will put issues at the crossroads of chronic disease and work, and of health care and occupational health on the agenda.

## Competing interests

The authors declare that they have no competing interests.

## Authors' contributions

FvD and IV were applicants on the funding proposal. IV has drafted the manuscript and is responsible for data collection. IV, GdV and AH were the main developers of the intervention and FvD was co developer. IV and FvD developed the study design. All authors have commented on draft versions of the manuscript and approved of the final manuscript.

## Pre-publication history

The pre-publication history for this paper can be accessed here:


